# Activation of TNFR2 sensitizes macrophages for TNFR1-mediated necroptosis

**DOI:** 10.1038/cddis.2016.285

**Published:** 2016-09-22

**Authors:** Daniela Siegmund, Juliane Kums, Martin Ehrenschwender, Harald Wajant

**Affiliations:** 1 Division of Molecular Internal Medicine, Department of Internal Medicine II, University Hospital Würzburg, Röntgenring 11, Würzburg 97070, Germany; 2 Institute of Clinical Microbiology and Hygiene, University Hospital Regensburg, Franz-Josef-Strauss-Allee 11, Regensburg 93053, Germany

## Abstract

Macrophages express TNFR1 as well as TNFR2 and are also major producers of tumor necrosis factor (TNF), especially upon contact with pathogen-associated molecular patterns. Consequently, TNF not only acts as a macrophage-derived effector molecule but also regulates the activity and viability of macrophages. Here, we investigated the individual contribution of TNFR1 and TNFR2 to TNF-induced cell death in macrophages. Exclusive stimulation of TNFR1 showed no cytotoxic effect whereas selective stimulation of TNFR2 displayed mild cytotoxicity. Intriguingly, the latter was strongly enhanced by the caspase inhibitor zVAD-fmk. The strong cytotoxic activity of TNFR2 in the presence of zVAD-fmk was reversed by necrostatin-1, indicating necroptotic cell death. TNFR1- and TNF-deficient macrophages turned out to be resistant against TNFR2-induced cell death. In addition, the cIAP-depleting SMAC mimetic BV6 also enforced TNF/TNFR1-mediated necroptotic cell death in the presence of zVAD-fmk. In sum, our data suggest a model in which TNFR2 sensitizes macrophages for endogenous TNF-induced TNFR1-mediated necroptosis by the known ability of TNFR2 to interfere with the survival activity of TRAF2-cIAP1/2 complexes.

Tumor necrosis factor (TNF) is a pleiotropic cytokine that occurs as a type II transmembrane protein but can be released from the plasma membrane by proteolytic processing.^1^ Membrane-bound and soluble TNF both contain the characteristic carboxy-terminal TNF homology domain, which is responsible for self-assembly into trimeric molecules and receptor binding. Membrane-bound and soluble TNF strongly interact with two receptors, TNFR1 and TNFR2, but the two forms of TNF are differentially effective in receptor activation.^1^ Whereas membrane-bound TNF activates TNFR1 and TNFR2 efficiently, soluble TNF is sufficient for TNFR1 activation but largely inactive upon binding to TNFR2. TNFR1 belongs to the death receptor subgroup of the TNF receptor family and can trigger apoptosis and necroptosis.^2, 3, 4^ However, cell death induction by TNFR1 is typically efficiently antagonized by concomitant activation of the cytoprotective classical NF*κ*B pathway and/or ubiquitous expression of anti-apoptotic proteins.^1, 2^ The latter involve FLIP proteins which generally inhibit death receptor-induced caspase-8 activation but also complexes containing TRAF2, cIAP1 and cIAP2 which specifically interfere with caspase-8 activation in context of TNFR1 signaling.^2, 3, 4^ Worth mentioning, TRAF2-cIAP1/2 complexes also mediate K63-linked ubiquitination of RIP1 in the TNFR1 signaling complex, thereby facilitating TNFR1-mediated activation of the classical NF*κ*B pathway. Indeed, TNFR1 signaling is predominantly pro-inflammatory as TNFR1-induced cell death is blocked as long as the aforementioned protective mechanisms are not impaired.

In contrast to TNFR1, TNFR2 contains no cytoplasmic death domain. Upon ligand binding, TNFR2 recruits TRAF2 and various TRAF2-associated proteins, such as TRAF1, cIAP1 and cIAP2, but also interacts with other signaling proteins independently of TRAF2.^1, 5^ TNFR2 activation has been linked to a variety of immune regulatory functions, which, in contrast to the activities of TNFR1, often result in anti-inflammatory effects.^6^

Murine models shed light on the complex interplay of the TNFR1–TNFR2 system *in vivo*, demonstrating additive, synergistic or even antagonistic effects. At the cellular level, several mechanisms for the cross-talk between TNFR1 and TNFR2 have been identified.^1^ Besides the obvious competition for ligand binding, TNFR1 and TNFR2 can induce, for example, autocrine TNF production in a cell type-specific manner.^1^ In context of TNFR1 activation by soluble TNF, subsequent induction of membrane-bound TNF results in costimulation of TNFR2, thereby converting the initially transient activation into sustained autocrine signaling. In addition, TNFR1 and TNFR2 compete for the cytoplasmic pool of TRAF2–cIAP1/2 complexes. By depletion and/or degradation of TRAF2, TNFR2 is capable to modulate TNFR1 signaling.^1^ Moreover, TNFR2 but not TNFR1, stimulates the alternative NF*κ*B pathway by triggering proteolytic processing of the inactive p100/RelB dimers into active p52/RelB NF*κ*B complexes.^7^ Notably, TNFR2-induced alternative NF*κ*B signaling can be enhanced by TNFR1-mediated induction of p100 and RelB expression via the classical NF*κ*B pathway.^7^

In macrophages, the complexity of the TNF-TNFR1/2 system is especially relevant. Macrophages on one hand co-express TNFR1 and TNFR2 and are on the other hand a pathophysiologically important source of TNF, for example, in response to a variety of pathogen-associated molecular patterns (PAMPs). TNF not only acts as a macrophage-derived effector molecule, but in an autocrine fashion also controls macrophage activation and survival, as seen for example during infection with mycobacteria.^8, 9, 10, 11, 12, 13, 14, 15, 16, 17^ However, the molecular mechanisms of TNF-induced cell death in macrophages are incompletely understood and were, therefore addressed in our study. Using macrophages genetically deficient for TNFR1, TNFR2 or TNF together with TNFR1- and TNFR2-specific TNF variants, we show that TNFR2 activation sensitizes macrophages for TNFR1-mediated necroptosis triggered by autocrine produced TNF and provide evidence that this is related to TNFR2-induced depletion/degradation of TRAF2-cIAP1/2 complexes.

## Results

### TNFR2 stimulation triggers necroptosis in zVAD-fmk treated macrophages

**Figure 1:**
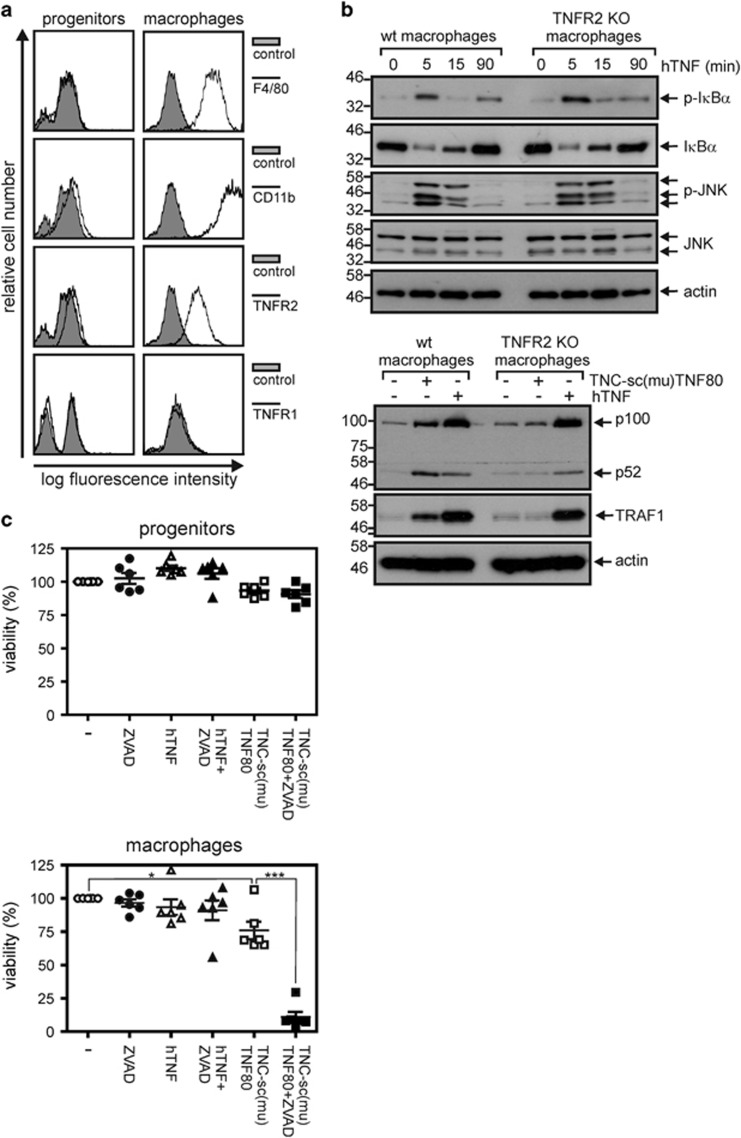
TNFR2 induces cell death in murine macrophages. (**a**) Hoxb8 immortalized murine MPCs and macrophages derived thereof were analyzed by flow cytometry for the indicated cell surface markers. (**b**) Macrophages were stimulated for the indicated times with 50 ng/ml human TNF or 200 ng/ml TNC-sc(mu)TNF80 and analyzed with respect to expression of the indicated proteins by western blotting of total cell lysates. Macrophages derived from HoxB8-immortalized MPCs of TNFR2-deficient mice served as an additional control for the TNFR2-indepenency of the observed effects. Positions of molecular weight markers (kDa) are indicated on the left. (**c**) MPCs and macrophages were stimulated with the indicated combinations of human TNF (50 ng/ml), TNC-sc(mu)TNF80 (200 ng/ml) and zVAD-fmk (20 *μ*M). After 36 h, cell viability was quantified using MTT assay or by crystal violet staining. Data points derived from 6 independent experiments together with mean±S.E.M. are depicted. *p<0.05; ***p<0.001

**Figure 2:**
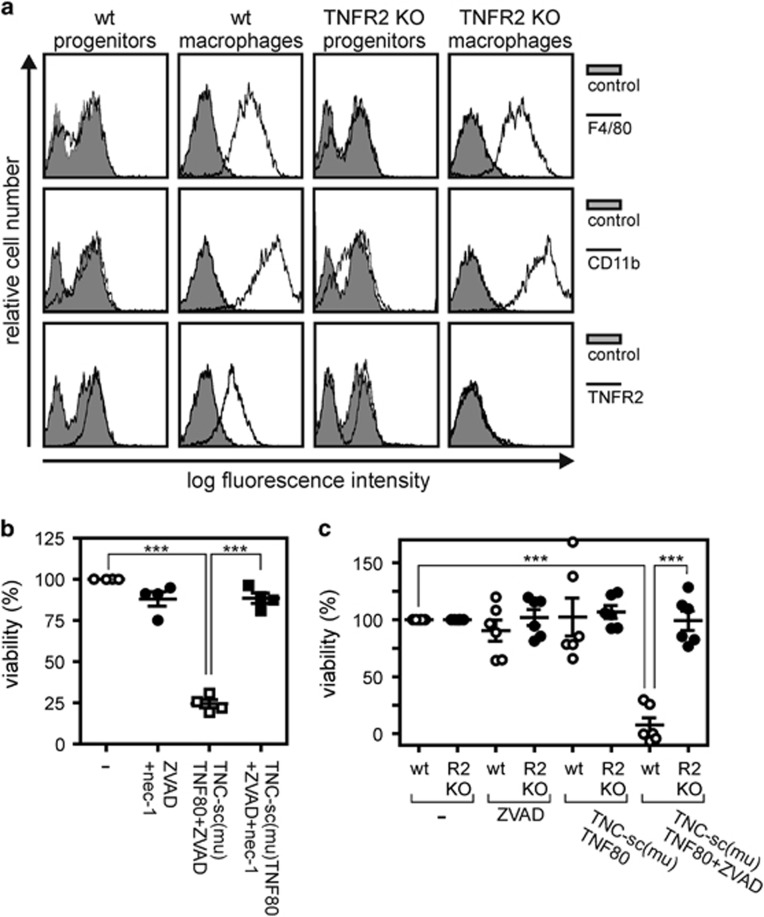
TNFR2 activation induces necroptosis in macrophages. (**a**) Wild type and TNFR2 knockout Hoxb8 immortalized MPCs and macrophages derived thereof were analyzed by flow cytometry for the indicated membrane proteins. (**b**) Macrophages derived from Hoxb8 immortalized MPCs were challenged with the indicated combinations of TNC-sc(mu)TNF80 (200 ng/ml), zVAD-fmk (20 *μ*M) and necrostatin-1 (45 *μ*M) for 36 h. Data points derived from four independent experiments together with mean±S.E.M. are depicted. (**c**) Wild type and TNFR2-deficient macrophages were stimulated as indicated with TNC-sc(mu)TNF80 (200 ng/ml) and ZVAD (20 *μ*M) and were analyzed after 36 h for their viability. Data points derived from six independent experiments together with mean±S.E.M. are depicted. ***p<0.001

For our study, we generated macrophages from HoxB8-immortalized murine myeloid progenitor cells (MPCs). Typical murine macrophage surface markers such as F4/80 and CD11b were detectable 5 days after differentiation into macrophages was initiated (Figure 1a). Flow cytometry revealed significant TNFR2 expression on macrophages, whereas there was only poor expression of this receptor on MPCs (Figure 1a). TNFR1 was not detectable by flow cytometry. However, functional studies with human TNF, which does not bind to murine TNFR2^18^ and consequently selectively stimulates TNFR1 in murine cells, revealed robust TNFR1 responsiveness as seen by activation of the classical NF*κ*B pathway and upregulation of the NF*κ*B target genes TRAF1 and p100 (Figure 1b). To determine the individual contribution of TNFR1 and TNFR2 to TNF-induced cell death of macrophages, we selectively stimulated the two receptors using soluble human TNF and a nonameric TNF fusion protein with specificity for TNFR2 (TNC-sc(mu)TNF80). Importantly, cells were also challenged with the TNFR1- and TNFR2-specific ligands in the presence of the pan-caspase inhibitor zVAD-fmk, which can unleash potential necroptotic activity by blocking caspase-8-mediated cleavage of RIP1.^19, 20^ Irrespective of zVAD-fmk, human TNF-mediated TNFR1 activation had no cytotoxic effects, neither in HoxB8-immortalized MPCs, nor in macrophages (Figure 1c). In macrophages, TNFR2 activation using TNC-sc(mu)TNF80 resulted in a mild cell death response, which, however, was strongly enhanced in the presence of zVAD-fmk (Figure 1c). In accordance with the fact that TNFR2 is not or only poorly expressed on murine MPCs (Figure 1a), TNC-sc(mu)TNF80 had no cytotoxic effect on this cell type (Figure 1c). Macrophages were rescued from the cytotoxic activity of the zVAD-fmk/TNC-sc(mu)TNF80 mixture by treatment with the RIP1 inhibitor necrostatin-1 (nec-1, ref. 21,21 Figures 2a and b). Since RIP1 has a crucial role in necroptotic signaling cascades this indicates that cell death induction by zVAD-fmk/TNC-sc(mu)TNF80 is due to necroptosis. Expectedly, TNC-sc(mu)TNF80 showed no cytotoxic effect on M-CSF differentiated SCF-ER-Hoxb8 immortalized macrophages derived of TNFR2-deficient mice (Figures 2a and c).

### TNFR2 stimulation triggers autocrine TNF-production and TNFR1-mediated necroptosis in murine macrophages

**Figure 3:**
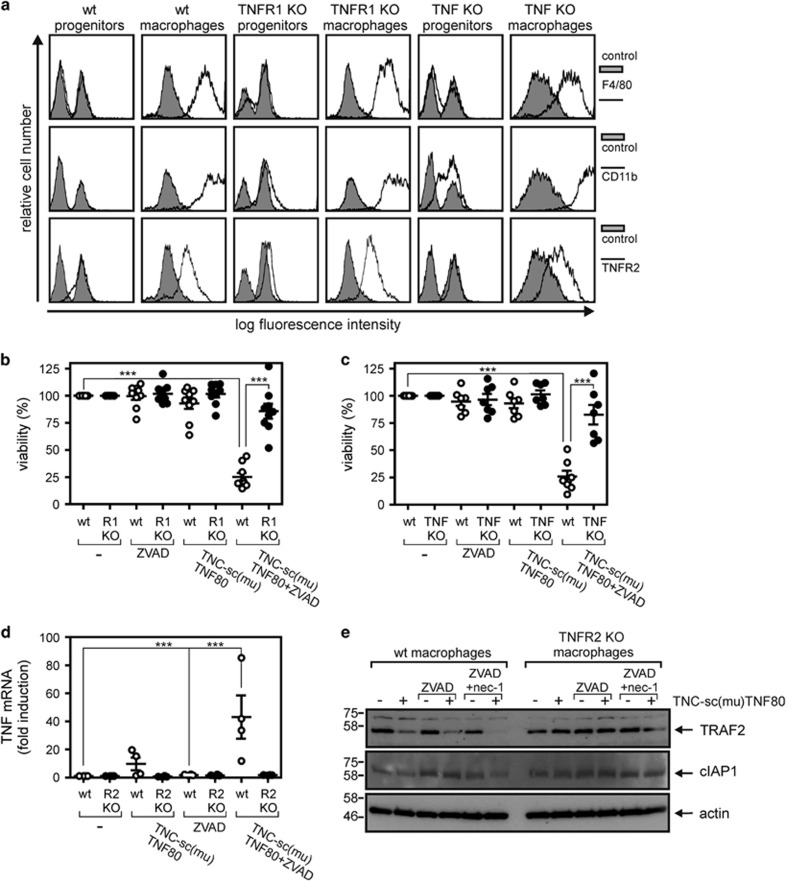
TNF and TNFR1 are required for zVAD-fmk/TNC-sc(mu)TNF80 induced cell death. (**a**) MPCs and macrophages derived thereof were analyzed by flow cytometry with for cell surface expression of the indicated proteins. (**b,c**) MPCs and macrophages derived from wild type, TNF- (b) and TNFR1-deficient mice (c) were stimulated with the indicated combinations of human TNF (50 ng/ml), TNC-sc(mu)TNF80 (200 ng/ml) and zVAD-fmk (20 *μ*M). After 36 hours cell viability was quantified using the MTT assay or crystal violet staining. Data points derived from 9 (**b**) or 7 (**c**) independent experiments together with with mean±S.E.M. are depicted. (**d**) Macrophages derived from HoxB8-immortalized MPCs of wild type and TNFR2-deficient mice were stimulated overnight with the indicated combinations of TNC-sc(mu)TNF80 (200 ng/ml) and zVAD-fmk (20 *μ*M). *Tnf* mRNA induction was analyzed by qPCR. Data points of four independent experiments together with mean±S.E.M. are shown. (**e**) Wild type and TNFR2-deficient macrophages were treated as indicated for 7 h with TNC-sc(mu)TNF80 (200 ng/ml), zVAD-fmk (20 *μ*M) and necrostatin-1 (45 *μ*M). Cells were analyzed by western blotting for the presence of TRAF2 and cIAP1. ***p<0.001

Notably, TNFR2 neither interacts with RIP1 nor with caspase-8, but has the ability to modulate the activity of these molecules in context of TNFR1 signaling by restricting the availability of TRAF2-cIAP1 and TRAF2-cIAP2 complexes.^1, 22, 23, 24^ We previously demonstrated in tumor cell lines that TNFR2-mediated depletion/degradation of cytoplasmic TRAF2-cIAP1/2 complexes not only antagonizes TNFR1-induced RIP1-mediated activation of the classical NF*κ*B pathway, but also enhances TNFR1-induced cell death.^23, 24^ Given that macrophages express TNF and that TNFR1 is a prominent and potent trigger of necroptosis,^2, 3, 4^ we evaluated whether TNF and TNFR1 are involved in induction of necroptosis by zVAD-fmk/TNC-sc(mu)TNF80. For this purpose, macrophages derived from Hoxb8-immortalized murine MPCs of TNF- and TNFR1-deficient mice (Figure 3a) were subjected to TNFR1/TNFR2 stimulation experiments. In both cell types, co-treatment with zVAD-fmk and TNC-sc(mu)TNF80 showed no significant cytotoxic effect (Figures 3b and c) indicating that TNFR2-induced necroptosis in macrophages is due to sensitization for endogenous TNF-induced TNFR1-mediated necroptosis. Moreover, exclusive stimulation of TNFR2 resulted in induction of TNF, which was even enhanced in the presence of zVAD-fmk (thus under necroptotic conditions) (Figure 3d). Together, these data raised the possibility that TNFR2/zVAD-fmk triggers TNFR1-mediated necroptosis in macrophages by inducing TNF production and concomitant interference with the inhibitory activity of TRAF2-cIAP1/2 complexes on RIP1-dependent necroptosis. Indeed, we found a strong reduction of TRAF2 levels after TNFR2 stimulation in the presence and absence of zVAD-fmk and/or nec-1 (Figure 3e). Notably, TRAF2 levels remained low in necrostatin-1-protected cells following zVAD-fmk/TNC-sc(mu)TNF80 challenge, indicating that the TNFR2-triggered loss of TRAF2 was not an epiphenomenon of ongoing necroptosis (Figure 3e). TNFR2 stimulation did not affect cIAP1 levels (Figure 3e). cIAP2 expression in macrophages is too low to allow detection by standard western blotting protocols.

### BV6 triggers TNF/TNFR1-mediated necroptosis in murine macrophages

**Figure 4:**
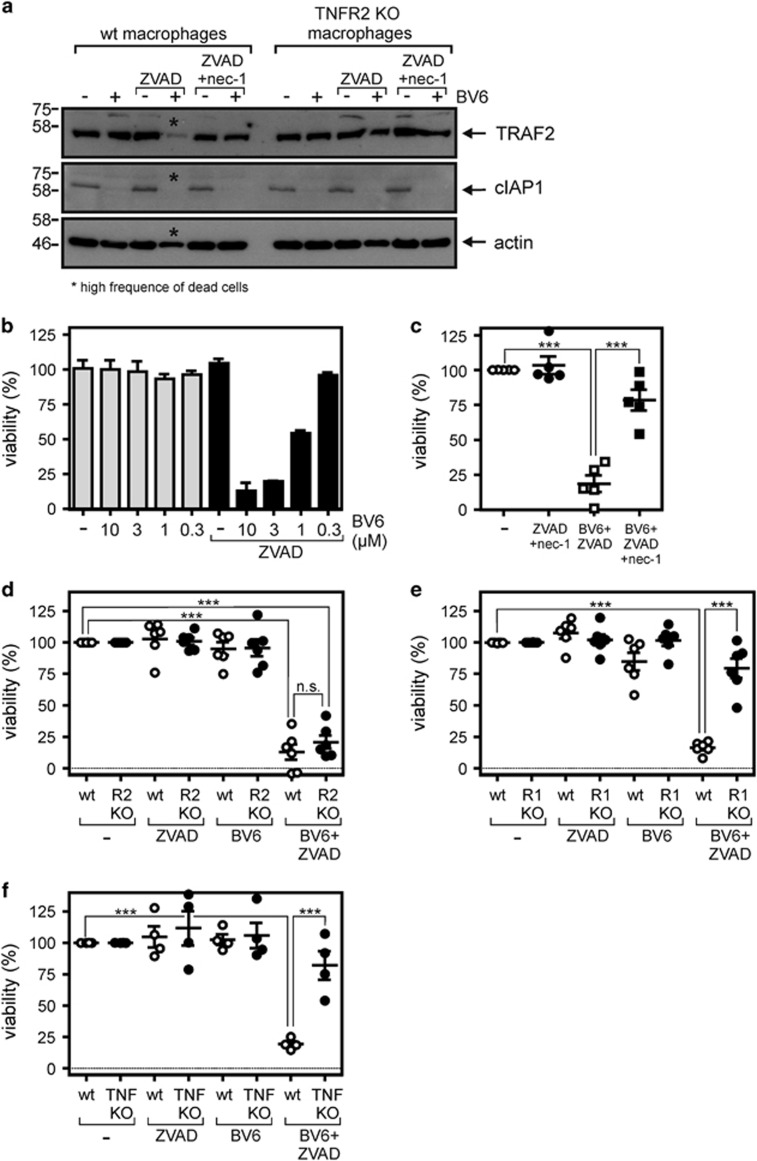
BV6 induces necroptosis in murine macrophages. (**a**) Wild type and TNFR2-deficient macrophages derived from HoxB8-immortalized MPCs were challenged for 7 h with the indicated combinations of BV6 (10 *μ*M), zVAD-fmk (20 *μ*M) and necrostatin-1 (45 *μ*M). Cells were finally analyzed by western blotting for the presence of TRAF2 and cIAP1. Please note, wild-type cells treated with BV6 and zVAD-fmk were already largely dead when cells were harvested for Western blot analysis. (**b**) Macrophages derived from HoxB8-immortalized MPCs were stimulated in triplicates (technical replicates) with the indicated concentrations of BV6 in the presence and absence of 20 *μ*M zVAD-fmk and analyzed for viability after 36 h. One of four representative experiments is shown. (**c**) HoxB8-immortalized MPC-derived macrophages were challenged with the indicated mixtures of 10 *μ*M BV6, 20 *μ*M ZVAD-fmk and 45 *μ*M necrostatin-1 and analyzed for viability after 36 h. Shown are data points with S.E.M. of five independent experiments. (**d–f**) Macrophages derived from Hoxb8 immortalized MPCs of wild type, TNF- (**d**), TNFR1- (**e**) and TNFR2-deficient mice (**f**) were stimulated with the indicated combinations of BV6 (10 *μ*M) and zVAD-fmk (20 *μ*M). After 36 hours cell viability was quantified using MTT assay or crystal violet staining. Data points derived from six (**d** and **e**) or four (**f**) independent experiments together with mean±S.E.M. are depicted. ***p<0.001

To further substantiate the idea that TNFR2 enhances TNFR1-induced necroptosis in macrophages by limiting the availability of TRAF2-cIAP complexes for TNFR1 stimulated by co-expressed TNF, we investigated the effects of the SMAC mimetic BV6 on macrophage viability. BV6 is a symmetric low-molecular weight compound that interacts with two molecules of XIAP, cIAP1 or cIAP2.^25^ In the case of cIAP1 and cIAP2, this results in dimerization-induced K48-linked auto-ubiquitination and subsequent proteasomal degradation.^25^ cIAP1 expression levels were severely reduced in BV6-treated macrophages irrespective of zVAD-fmk or necrostatin-1 treatment, while there was no effect on TRAF2 (Figure 4a). In line with an anti-necroptotic activity of TRAF2–cIAP complexes (and similar to TNFR2 stimulation), BV6 triggered cell death in macrophages in the presence of zVAD-fmk in a dose-dependent manner (Figure 4b). zVAD-fmk/BV6-induced cytotoxicity was largely abolished by necrostatin-1 treatment (Figure 4c), again indicating necroptotic cell death. Moreover, zVAD-fmk/BV6-induced necroptosis was abrogated in macrophages derived from Hoxb8-immortalized murine MPCs of TNF or TNFR1 knockout mice, but remained unaffected in TNFR2 knockout macrophages (Figures 4d and f). This strengthened the idea that zVAD-fmk/BV6-induced necroptosis finally emanates from the TNF/TNFR1 axis. Collectively, our data suggest an anti-necroptotic role of TRAF2-cIAP complexes, along with capsase-8, in macrophages to prevent suicidal autocrine TNF-TNFR1 signaling.

### TNFR2 is involved in LPS-induced necroptosis in murine macrophages

**Figure 5:**
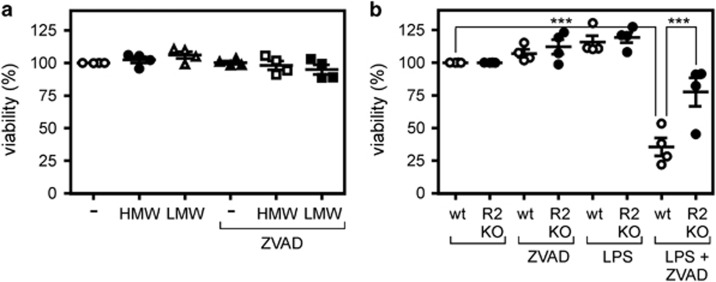
Effect of TLR3 and TLR4 activation on macrophage viability in the presence and absence of zVAD-fmk. (**a,b**) Macrophages derived of Hoxb8 immortalized murine MPCs were stimulated with 2 *μ*g/ml of high-molecular weight or low-molecular weight poly(I:C) (= TLR3 activation) (**a**) or LPS (1 *μ*g/ml,=TLR4 activation) (**b**) in the presence and absence of zVAD-fmk (20 *μ*M). After 36 h, cell viability was quantified using the MTT assay. Data points derived from four independent experiments together with mean±S.E.M. are depicted. ***p<0.001

TLR3 and TLR4 are not only major inducers of TNF production in macrophages but have also the potential to trigger necroptosis in this cell type.^26, 27, 28, 29^ To evaluate whether TNFR2 has a role in TLR-induced necroptosis of macrophages, we performed initial experiments with agonists of TLR3 and TLR4. Although the TLR3 agonist poly(I:C) showed no cytotoxic activity in macrophages differentiated from HoxB8-immortalized MPCs, TLR4 stimulation with LPS in the presence zVAD-fmk resulted in robust cell death (Figures 5a and b). This cytotoxic effect was severely reduced in TNFR2 knockout macrophages indicating a role of the TNF system in TLR4-induced necroptosis. At the first glance, this is contradictory to a former publication arguing for TNF-independent necroptosis by LPS in macrophages,^27, 28, 29^ but another publication reported partly reduced zVAD/LPS-induced cell death.^30^ Moreover, a recent study demonstrated that the role of TNF in LPS-induced necroptosis in macrophages is dependent on the LPS concentration used for stimulation (high-concentrations TNF-independent, low-concentrations TNF-dependent).^31^

## Discussion

Macrophages are perhaps the best studied primary cell type capable to undergo necroptotic cell death. Indeed, macrophages are strongly responsive to the majority of natural inducers of necroptosis including TNF, interferons and various viral and bacterial PAMPs.^26, 27, 32, 33^ Macrophages activated by PAMPs, for example, LPS, are an integral part of the host's first line of defense against invading pathogens. Some bacteria evade macrophage control by inducing cell death of infected macrophages. This occurs by various mechanisms reaching from caspase-dependent apoptosis over IL-1β-induced pyroptosis to RIP1/RIP3-mediated necroptosis.^34^ Bacteria-triggered necroptosis in macrophages has been demonstrated for various bacteria producing pore forming toxins such as *Staphylococcus aureus*, *Listeria monocytogenes*, uropathogenic strains of *Eschericha coli* and *Salmonella enterica* serovar Typhimurium (*S. Typhimurium*).^27, 35, 36^ Although the pore forming toxin producing bacteria trigger necroptosis by membrane disruption and ion dysregulation, *S. Typhimurium*-induced macrophage necroptosis was attributed to IFNβ induction and TNFR1/R2-independent RIP1/RIP3-mediated necroptosis.^27^ The latter is in line with studies demonstrating TLR3- and TLR4-induced necroptosis of macrophages.^28, 29, 37^ Despite the prototypic nature of TNF-induced necroptosis and the overwhelming role of the TNF-TNFR system in macrophage biology, the potential of macrophages to undergo TNF-induced necroptosis has not been evaluated yet in detail. Especially, the specific role of the two TNF receptors in TNF-induced necroptosis in macrophages remained unclear. We, therefore, elucidated in our study the interplay of endogenous TNF with TNFR1 and TNFR2 as well as the individual contribution of the two TNF receptors to TNF-induced necroptosis in murine macrophages.

Our initial observation that exclusive exogenous triggering of TNFR2 in the presence of the caspase-inhibitor zVAD-fmk is fully sufficient to induce robust necroptotic cell death in murine macrophages (Figures 1c and 2) was at the first glance surprising. TNFR2, in contrast to TNFR1, does not interact with RIP1 and has also not been recognized so far as a trigger of necroptotic cell death, although a ‘deadly potential' of TNFR2 has been observed before. More than a decade ago, we and others demonstrated that a very few tumor cell lines undergo apoptosis in response to TNFR2 stimulation due to endogenous TNF production and subsequent TNFR1 activation.^38, 39^ We, therefore, analyzed the involvement of endogenous TNF and TNFR1 in macrophage necroptosis following TNFR2 activation. Indeed, it turned out that (i) TNFR2 induces TNF under necroptotic conditions in macrophages (Figure 3d) and (ii) that both TNF and TNFR1 deficiency protects against TNC-sc(mu)TNF80/zVAD-fmk-triggered necroptosis (Figures 3b and c), indicating that autocrine TNF/TNFR1 signaling is also operative in this scenario. Although the results from the TNF- and TNFR1-deficient macrophages nicely fit into the concept that TNFR2 kills macrophages in the presence of zVAD-fmk by induction of TNF and subsequent triggering of TNFR1-mediated necroptosis, this straightforward model cannot be complete. The crucial unanswered question is why selective exogenous stimulation of TNFR1 is not sufficient to trigger macrophage necroptosis in a similar fashion as TNFR2-induced endogenous TNF. In this context, it is important to note that the discussed apoptosis-inducing activity of TNFR2 is not only based on TNF-induction with subsequent TNFR1 activation but also needs a second, *per se* non-toxic mechanism sensitizing for TNFR1-induced apoptosis.^38, 40^ The ability of TNFR2 to sensitize for TNFR1-induced apoptosis represents a general TNFR1–TNFR2 cross-talk mechanism and is not only of relevance in cells where triggering of TNFR2 alone results in cell death but also in cell lines and cell types where TNFR2 stimulation alone is not apoptotic.^23, 24, 41, 42, 43, 44, 45, 46^ Indeed, a sensitizing effect of TNFR2 on TNFR1-induced cell death has also already been observed in cells undergoing TNFR1-induced necrosis.^47, 48^ Thus, the superior necroptosis-triggering activity of TNFR2 might indicate that this sensitizing mechanism is of special relevance for TNF-induced cell death in macrophages. At the molecular level, TNFR2-induced enhancement of TNFR1-induced apoptosis has been traced back to the ability of TNFR2 to deplete/degrade TRAF2-cIAP1 and TRAF2-cIAP2 complexes which in context of TNFR1 signaling inhibit complex II-mediated caspase-8 activation. Since TRAF2 and the cIAPs also inhibit necroptosis, depletion and/or degradation of these molecules may also crucially contribute to zVAD-fmk/TNFR2-induced necroptosis in macrophages. In accordance with this idea, we observed significant reduction of TRAF2 levels after TNFR2 stimulation (Figure 3e).

cIAPs fulfill in macrophages an anti-necroptotic function. This has already been recognized upon treatment with the SMAC mimetics/IAP antagonists SM-164 and compound A.^49, 50^ The effects of SMAC mimetics in macrophages resemble those described for TNFR2 activation in several ways as (i) both trigger degradation of cIAP1 and cIAP2, thereby sensitizing cells for TNFR1-induced cell death and (ii) both activate transcription factors of the NF*κ*B family enabling (in a cell-type dependent manner) autocrine TNF production. In line with this notion, we found that a mixture of zVAD-fmk and BV6, but not BV6 alone, triggered TNF- and TNFR1-dependent necroptosis (Figures 4d and e). In accordance with the fact that BV6 and TNFR2 control TNFR1-induced necroptosis at the level of TRAF2 and the cIAPs, TNFR2 deficiency showed no effect on zVAD-fmk/BV6-induced necroptosis (Figure 4f). Noteworthy, triggering of macrophage necroptosis by LPS, which stimulates TNF production, also revealed a TNFR2-dependent component (Figure 5b) suggesting that there is crosstalk between TNF induction and direct TLR4-triggered TRIF-mediated necroptosis. Taken together our data suggest that TNFR2, due to its ability to deplete TRAF2-cIAP complexes, is a (patho)physiological modulator of TNFR1-induced necroptosis in macrophages.

## Materials and Methods

### Compounds, antibodies and reagents

The SMAC mimetic BV6 was synthesized as described in ref. 51 on order by Syngene (Bangalore, India). Antibodies used were purchased from Cell Signaling Technology Beverly, MA, USA (anti-p100/p52, rabbit polyclonal IgG, # 4882; anti-I*κ*B*α* mouse IgG1, clone L35A5, # 4814; anti-phospho-I*κ*B*α* (Ser32) rabbit mAb IgG, clone 14D4, # 2859; anti-TRAF2, rabbit polyclonal IgG, # 4724), Miltenyi Biotec, Bergisch Gladbach, Germany (anti-CD11b rat IgG2b-PE, clone M1/70.15.11.5, # 130-091-240; rat IgG2b-PE, # 130-102-663; anti-mouse F4/80 human IgG1, clone REA126, # 130-102-943; REA control antibody-PE, clone REA239, # 130-104-613; anti-mouse TNF rat IgG1-PE, clone MP6-XT22, # 130-102-386; anti-mouse TNFR2 human IgG1-PE, clone REA228, # 130-104-697), Santa Cruz Biotechnology Santa Cruz, CA, USA (anti-mouse TNFR1 hamster mAb IgG-PE, clone 55R-170, # sc-12746; hamster IgG-PE, sc-2875), Enzo Life Sciences, NY, USA (anti-cIAP1 rat IgG2a, clone 1E1-1-10, # ALX-803-335) and Affymetrix eBioscience (rat IgG1-PE, # 12-4301-83). zVAD-fmk was obtained from Thermo Fisher Scientific Waltham, MA, USA (Bachem # N-1510.0025) and necrostatin-1 from Biomol, Hamburg, Germany (# AP-309). For stimulation of TLR3 and TLR4, high- and low-molecular weight poly(I:C) and LPS form the mouse TLR1-9 agonist kit (# tlrl-kit1mw) from InvivoGen (San Diego, Ca, USA) have been used. Human TNF was a kind gift from Prof. Daniela Männel (University of Regensburg, Germany). The strongly agonistic murine TNFR2-specific nonameric murine TNF variant TNC-sc(mu)TNF80 was produced and characterized as described elsewhere (under revision elsewhere). To control TNC-sc(mu)TNF80 for its LPS content, the latter was determined with the Pierce LAL Chromogenic Endotoxin Quantitation Kit (Thermo Fisher Scientific) as described in the manual of the manufacturer. If necessary LPS has been removed using the Pierce High Capacity Endotoxin Removal Resin packed in columns according to the recommendations of the supplier. Afterwards samples were controlled for successful LPS removal.

### Generation of SCF-ER-Hoxb8 immortalized murine MPCs and their differentiation into macrophages

Hoxb8 immortalized murine MPCs were generated essentially as described elsewhere.^52^ In brief, C57Bl/6 mice (wild type, TNFR1 knockout, TNFR2 knockout or TNF knockout) were sacrificed in accordance with animal welfare. Femurs were cut and flushed with icecold PBS. After filtration (100 *μ*m pores) on ice, cells were pelleted (8 min, 4 °C, 400 g) and resuspended in 5 ml ammonium sulfate solution (150 mM NH_4_Cl, 0.1 mM Na_2_EDTA, 1 mM KHCO_3_, pH 7.4) to lyse red blood cells. Non-lysed cells were separated by additional centrifugation (8 min, 4 °C, 400 g), washed tree times with ice-cold PBS and cultivated for 2 h in 10 ml RPMI 1640 medium supplemented with 10% (v/v) FCS and 30 *μ*M ß-mercaptoethanol to allow adherence of fibroblasts. The supernatant with non-adherent cells was then transferred to a new cell culture disc and supplemented with 10 ng/ml murine IL-3 (Miltenyi Biotec), 10 ng/ml murine IL-6 (Miltenyi Biotec), 4% (v/v) conditioned cell culture supernatant of SCF producing CHO cells and additional 5% (v/v) FCS. After 2 days cells were used for transduction (2 × 10^6^ in 500 *μ*l in 1% Optimem, 10% FCS, 30 *μ*M ß-mercaptoethanol) with 3 ml HoxB8 encoding virus supernatant (DMEM-Glutamaxx (Thermo Fisher Scientific), 10% (v/v) FCS). A plasmid for estrogen-regulated HoxB8 expression (3HAERHBH-HoxB8) was kindly provided by Hans Haecker (St. Jude Children's Research Hospital, Memphis, USA). After 3 h, 3 ml Optimem, 10% (v/v) FCS, 1% (v/v) L-glutamine, 30 *μ*M ß-mercaptoethanol with 1 *μ*M estradiol and 4% (v/v) SCF containing cell culture supernatant were added. Three days later, 2 ml supernatant were exchanged with RPMI 1640 medium with 10% (v/v) FCS, 1% (v/v) L-glutamine and 30 *μ*M ß-mercaptoethanol. After additional 3 days, 3 ml of cell culture were transferred into a new well and supplemented with 3 ml of fresh medium. Medium was changed three times per week and every time freshly supplemented with 1 *μ*M estradiol (Sigma, Steinheim, Germany) and 4% (v/v) conditioned cell culture supernatant of SCF producing CHO cells. To induce differentiation into macrophages, the progenitor cells were washed three times with warm PBS supplemented with 10% (v/v) FCS and seeded on uncoated cell culture plates or flasks in RPMI 1640 medium supplemented with 10% (v/v) FCS, 1% (v/v) L-glutamine, 30 *μ*M ß-mercaptoethanol and 10% (v/v) conditioned cell culture supernatant of M-CSF producing L929 cells. Cell culture media was replaced after 3 days. Five days after initiating the differentiation, cells were harvested. An aliquot was analyzed using flow cytometry to control the successful differentiation into macrophages. The remaining cells were seeded for the experiments into 96-well (45 × 10^3^ per well) or 6-well plates (1 × 10^6^ per well).

### FACS analysis

To detect cell-surface expressed proteins, cells were harvested and resuspended in PBS. PE-labeled antibodies of the specificity of interest and corresponding isotype control antibodies were added at the dilution recommended by the supplier for 0.5 h on ice. After removal of unbound antibodies (3 × wash with PBS) cells were analyzed with a FACSCalibur (BD Biosciences, Heidelberg, Germany) following standard procedures.

### Western blotting

MPCs were seeded in 6-well plates and were differentiated to macrophages. The latter were then subjected to stimulation with the reagents of interest and whole cell lysates were prepared. For this purpose, the cells were scraped into ice-cold PBS with a rubber policeman, pelleted by centrifugation and after resuspension in 4 × Laemmli sample buffer (8% (w/v) SDS, 0.1 M dithiothreitol, 40% (v/v) glycerol, 0.2 M Tris, pH 8.0) supplemented with phosphatase inhibitor cocktail II (Sigma) lysed by sonification (ten pulses) and heating for 5 min at 96 °C. Remaining insoluble debris was removed by centrifugation and the resulting whole cell lysates were subjected to SDS–PAGE. Separated proteins were transferred from the gel to nitrocellulose and after blocking of nonspecific binding sites by incubation in Tris-buffered saline with 0.1% (v/v) Tween 20 and 5% (w/v) dry milk, immunoblotting was performed with the primary antibodies of interest, horseradish peroxidase-conjugated secondary antibodies (Dako, Hamburg, Germany) and the ECL western blotting detection reagents and analysis system (Thermo Fisher Scientific).

### qPCR

Total RNA was isolated using the RNeasy mini kit from Qiagen (Valencia, CA, USA) according to the protocol of the manufacturer. Two micrograms of total RNA were transcribed into complementary DNA using the high-capacity cDNA reverse transcription kit (Applied Biosystems, Carlsbad, CA, USA). *Tnf* mRNA levels were quantified using the TaqMan mouse *Tnf* (Mm00443258_m1) gene expression assay (Applied Biosystems) and an ABI Prism 7900 sequence detector (Applied Biosystems). qRT-PCR reactions were performed in triplicates for each sample of an experiment and normalized to the housekeeping gene *Hprt1* (Mm00446968_m1). mRNA levels were calculated using the SDS 2.1 software (Applied Biosystems).

### Evaluation of cellular viability

MPCs or macrophages derived thereof (2 × 10^4^/well) were cultivated overnight in 100 *μ*l culture medium in 96-well plates and stimulated the next day in triplicates with the reagents of interest. After 36 h cell viability was determined using the MTT assay or crystal violet staining. For normalization of viability each experiment not only included untreated cells, but also cells that had been treated with a cytotoxic mixture (1 *μ*g/ml Fc-CD95L, 20 *μ*M CHX, 1% (w/v) sodium azide) triggering maximal cell death. The average of the triplicate of cells treated with the cytotoxic mixture was defined as 0% viability and the average of the triplicate of the untreated cells was set to 100% viability. Each measurement was finally normalized according to these values and the averages of the various triplicates were considered as a data point for further analyses using the One-way ANOVA Bonferroni's multiple comparison test function of the GraphPad Prism5 software (GraphPad software, La Jolla, CA, USA).

## Abbreviations

cIAP1/2: cellular inhibitor of apoptosis-1/2
LPS: lipopolysaccharide
MPC: murine myeloid progenitor cells
RIP1: receptor interacting protein
PAMP: pathogen-associated molecular pattern
TLR3/4: Toll-like receptor 3/4
TNFR1/2: tumor necrosis factor (TNF) receptor 1/2
zVAD-fmk: N-benzyloxycarbonyl-Val-Ala-Asp(O-Me) fluoromethyl ketone
